# Risk of Contralateral Hip Fracture Following Initial Hip Fracture Among Geriatric Fragility Fracture Patients

**DOI:** 10.5435/JAAOSGlobal-D-23-00001

**Published:** 2023-07-07

**Authors:** Philip P. Ratnasamy, Katelyn E. Rudisill, Oghenewoma P. Oghenesume, Matthew D. Riedel, Jonathan N. Grauer

**Affiliations:** From the Department of Orthopedics & Rehabilitation, Yale School of Medicine, New Haven, CT.

## Abstract

Geriatric hip fractures are common and associated with high morbidity and mortality. This study aimed to evaluate the incidence, timing, and risk factors of contralateral hip fracture after initial hip fracture.

**Methods:**

Initial hip fractures in patients older than 65 years were abstracted from the national, administrative M91Ortho PearlDiver data set. Incidence and timing of contralateral hip fractures in the following 10 years were identified. Kaplan-Meier survival analysis until contralateral hip fracture was conducted. To account for patient mortality over the later years, 2-year univariate and multivariate analyses were used to determine factors predictive of contralateral hip fracture.

**Results:**

Of the initial 104,311 hip fractures identified, contralateral hip fracture in the 10 years that followed was identified for 7,186 (6.9%), of which 68.4% occurred in the first 2 years. Kaplan-Meier survival analysis until contralateral fracture revealed the 10-year incidence to be 12.9% when controlling for those lost from the data set during the study period. Multivariate logistic regression revealed independent predictors of contralateral hip fracture in the 2 years after index hip fracture, when the incidence was greatest, to be female sex (odds ratio [OR] 1.15), body mass index < 20 (OR 1.30), and percutaneous pinning surgery for initial hip fracture fixation (OR 1.58) (*P* < 0.0001 for each).

**Conclusions:**

In a national cohort of 104,311 geriatric hip fractures, Kaplan-Meier analysis for contralateral hip fracture found the 10-year incidence to be 12.9%, of which nearly 70% were in the first 2 years, and predisposing factors were defined. As such, future research should aim to identify the cause and mitigate the risk of secondary contralateral hip fractures in geriatric patients.

Geriatric hip fractures are a major public health concern—with approximately 300,000 cases per year in the United States.^1^ The morbidity after such injuries is high,^2,3^ and associated costs are a burden to the health system.^4,5^ This patient population may be at increased risk of contralateral hip fracture due to falls, bone quality, deconditioning, and other factors.

Previous studies have reported the risk of a subsequent contralateral hip fracture between 6% and 9%,^6,7^ with average estimates for lifetime risk of secondary hip fractures reported at approximately 20%.^8^ These previous studies had several limitations. A 2006 study by Nymark et al^6^ was geographically limited, only studying patients from Funen County, Denmark, while a study by Schroder et al^7^ only identified 256 patients with contralateral hip fracture, limiting analysis of predictive factors for secondary fracture. A meta-analysis by Egan et al^8^ comprised randomized controlled trials, which were limited by less than 2 years of follow-up after initial hip fracture^9,10,11,12^ and sample sizes of less than 400 patients.^9,10,11,12^ These trials controlled for the effect of different factors on the risk of subsequent hip fracture, including pharmacotherapy,^9,10^ inpatient rehabilitation,^11^ and hip protectors.^12^ Inherent to randomized controlled trials, such studies had restricted external validity.^9,10,11,12^

Patients who sustain a contralateral hip fracture are subject to markedly worse postoperative results, with increased complications, morbidity, and mortality compared with initial fractures.^13^ One study examining 288 patients with geriatric hip fracture found that those being treated for a second hip fracture were markedly more likely to have reduced mobility, be rehospitalized, and experience mortality compared with those being treated for a first hip fracture.^14^ Another study similarly reported markedly greater 1-year mortality in patients who experience a second hip fracture compared with patients with primary hip fracture.^15^ Initiatives exist to organize global efforts to reduce the burden of secondary hip fractures (and other fractality fractures), such as the American Society for Bone and Mineral Research Secondary Fracture Prevention Initiative^16^; however, better characterization of predictive risk factors and associations would help develop perioperative guidelines to prevent these occurrences effectively.^17^

Given that present data regarding contralateral hip fractures are limited by geographic location and scope of study, this study aimed to better elucidate the incidence, timing, and risk factors of contralateral hip fracture in the 10 years after initial hip fracture using a large, national health administrative data set. Through such analysis, providers may be better equipped to modify care pathways and patient education to reduce the risk of subsequent hip fracture, particularly in those patients at greatest risk.

## Methods

### Database and Cohort

Data for this retrospective cohort study were derived from the PearlDiver M91Ortho data set: a large Health Insurance Portability and Accountability Act–compliant health administrative database containing information on nearly 91 million orthopaedic patients in the United States. The PearlDiver database, maintained by PearlDiver Technologies, contains billing claims data across all specialty types, payer types, and sites of care across all geographic regions in the United States. After deidentification, PearlDiver Technologies inputs all collected billing claims information into the database for extraction and analysis. This is a well-validated data set used in numerous previously published studies. All PearlDiver data are aggregated and deidentified. As such, our Institutional Review Board granted studies using this database exemption from review.

Inclusion criteria were patients older than 65 years who experienced hip fracture. This was identified by the first incidence of hip fracture fixation surgery (current procedural terminology [CPT] codes CPT-27130, CPT-27125, CPT-27235, CPT-27236, CPT-27244, and CPT-27245) after hip fracture diagnosis (established by International Classifications of Disease [ICD] codes).

Patient demographic factors were extracted and tabulated, including age, sex, Elixhauser Comorbidity Index (ECI, a well-validated longitudinal measure of patient comorbidity burden calculated using ICD 9 and 10 codes),^18^ body mass index (BMI; kg/m^2^, <20, 20-20/not specifically defined, and 30+), and type of fixation surgery used to treat initial hip fracture (percutaneous pinning [CPT-27235], side plate/intramedullary hip screw [CPT-27244 and CPT-27245], hemiarthroplasty [CPT-27125 and 27236], and total hip arthroplasty [CPT-27130]). Total hip arthroplasty was only included if conducted for the primary diagnosis of hip fracture within the data set.

### Incidence of Contralateral Hip Fracture

Contralateral hip fractures within 10 years of initial hip fracture were determined by identifying subsequent incidences of hip fracture fixation surgery (CPT-27130, CPT-27125, CPT-27235, CPT-27236, CPT-27244, and CPT-27245) among the cohort of patients who had already once underwent hip fracture fixation surgery. The total number of 10-year contralateral hip fractures and yearly incidence of contralateral hip fractures were determined using these data.

Because the selected patient population was 65 years or older and experiencing hip fracture (a pathology of high morbidity and mortality in geriatric populations), a 10-year Kaplan-Meier survival curve with an end point of contralateral hip fracture was constructed to account for patients dropping out of the database because of death or other factors. This curve provided a better representation of the actual risk of contralateral hip fracture in the 10 years after initial hip fracture. Notably, the PearlDiver system–designated patients have dropped out of the database if they did not have any billing claims information for 1 contiguous year.

### Data Analysis

Given that most of the contralateral hip fractures occurred in the first 2 years after initial hip fracture, univariate analysis was used to compare characteristics of patients with hip fracture who did and did not experience contralateral hip fracture within 2 years of their initial hip fracture. Differences in patient sex, BMI, and initial hip fracture fixation surgery type were compared using Pearson chi square analysis. Differences in patient age and ECI were compared using the Welch *t-test*.

Multivariate logistic regression analysis was then conducted to determine independent predictors of contralateral hip fracture within 2 years of initial hip fracture. Odds ratios (ORs) and 95% confidence intervals were calculated for each category analyzed and compared with their respective referent categories.

All statistical analyses were conducted using the PearlDiver system—with statistical significance reached at *P* < 0.05. Prim9 (GraphPad Softwares) and Microsoft Excel (Microsoft) were used to create all figures.

## Results

### Study Cohort and Incidence of Contralateral Hip Fractures

**Figure 1:**
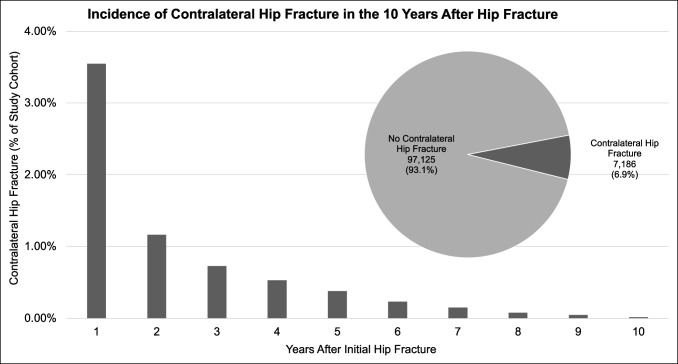
Bar graph showing the yearly incidence of contralateral hip fractures in the 10 years after initial hip fracture. Pie chart illustrating the overall incidence of 10-year contralateral hip fractures.

After excluding all patients younger than 65 years and any with a diagnosis of infection or malignancy on the day of their initial hip fracture diagnosis, 104,311 patients with initial hip fracture were identified by the primary occurrence of hip fracture surgery. Of this cohort, 7,186 patients (6.9%) experienced contralateral hip fracture within the following 10 years (Figure 1).

Of all contralateral hip fractures that occurred within 10 years of initial hip fracture, 68.4% occurred within the first 2 years. In the first year, 3,699 patients (3.55% of the total study cohort) experienced contralateral hip fracture, followed by 1,214 (1.16%) in the second year. By the third year after initial hip fracture, 761 (0.73%) experienced a contralateral hip fracture, with a continued drop through year 10, where only 19 patients (0.02%) had a contralateral hip fracture.

**Figure 2:**
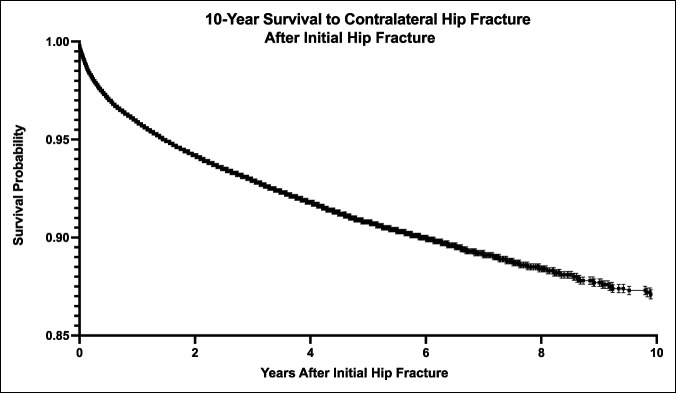
Graph showing 10-year Kaplan-Meier survival analysis to contralateral hip fracture after initial hip fracture.

Kaplan-Meier analysis was then conducted to determine the 10-year survival to contralateral hip fracture when accounting for loss of follow-up in the database because of patient death or other factors (Figure 2). This analysis revealed that 87.1% (standard error 0.20%) of patients with hip fracture had not experienced a contralateral hip fracture by 10 years, suggesting a real 10-year incidence rate of contralateral hip fracture of 12.9%.

### Factors Associated With Contralateral Hip Fracture

Demographic characteristics of all patients who experienced an initial hip fracture are presented in Table 1. The total study cohort had an average ± SD age of 73.91 ± 2.54 years and was female predominant (71.4%). The cohort had high ECI (5.25 ± 3.6) in general, and most patients fell into the BMI 20-29/not specifically defined category (88.6%). The most common initial hip fracture fixation procedure conducted was side plate/intramedullary hip screw (48.4%), followed by hemiarthroplasty (40.4%).

**Table 1:** Univariate Analysis of Characteristics of Patients With Hip Fracture

Factor	Total	No 2-Year Contralateral Hip Fracture	2-Year Contralateral Hip Fracture	*P*
N	104,311 (100%)	99,398 (95.3%)	4,913 (4.7%)	
Age (mean ± SD)	73.91 ± 2.54	73.91 ± 2.55	74.40 ± 2.55	
65-69	6,460 (6.2%)	6,171 (6.2%)	289 (5.9%)	<0.0001
70-74	52,221 (50.1%)	49,693 (50%)	2,528 (51.5%)	
75-79	44,262 (42.4%)	42,196 (42.5%)	2,066 (42.1%)	
80+	1,368 (1.3%)	1,338 (1.3%)	30 (0.6%)	
Sex				
Female	74,529 (71.4%)	70,884 (71.3%)	3,645 (74.2%)	<0.0001
Male	29,782 (28.6%)	28,514 (28.7%)	1,268 (25.8%)	
ECI (mean ± SD)	5.25 ± 3.6	5.25 ± 3.58	6.62 ± 3.8	
0-3	37,938 (36.4%)	36,148 (36.4%)	1,790 (36.4%)	<0.0001
4-6	32,802 (31.4%)	31,236 (31.4%)	1,566 (31.9%)	
7-9	20,100 (19.3%)	19,160 (19.3%)	940 (19.1%)	
10+	13,471 (12.9%)	12,854 (12.9%)	617 (12.6%)	
BMI (kg/m^2^)				
<20	7,100 (6.8%)	6,675 (6.7%)	425 (8.7%)	<0.0001
20-29/not specifically defined	92,396 (88.6%)	88,121 (88.7%)	4,275 (87%)	
30+	4,815 (4.6%)	4,602 (4.6%)	213 (4.3%)	
Fixation type				
Pinning	8,623 (8.3%)	8,023 (8.1%)	600 (12.2%)	<0.0001
Side plate/Intramedullary hip screw	50,528 (48.4%)	48,334 (48.6%)	2,194 (44.7%)	
Hemiarthroplasty	42,169 (40.4%)	40,165 (40.4%)	2,004 (40.8%)	
Total Hip Arthroplasty	2,991 (2.9%)	2,876 (2.9%)	115 (2.3%)	

BMI = body mass index, ECI = Elixhauser Comorbidity Index

Table 1 also presents the demographics of patients who did and did not experience contralateral hip fracture within 2 years of their initial hip fracture. Demographics were determined for this interval because nearly 70% of contralateral hip fractures occurred in the first 2 years after initial hip fracture. On univariate analysis, all characteristics were markedly correlated with risk of contralateral hip fracture (*P* < 0.0001 for age, sex, ECI, BMI, and initial hip fracture fixation surgery type).

**Figure 3:**
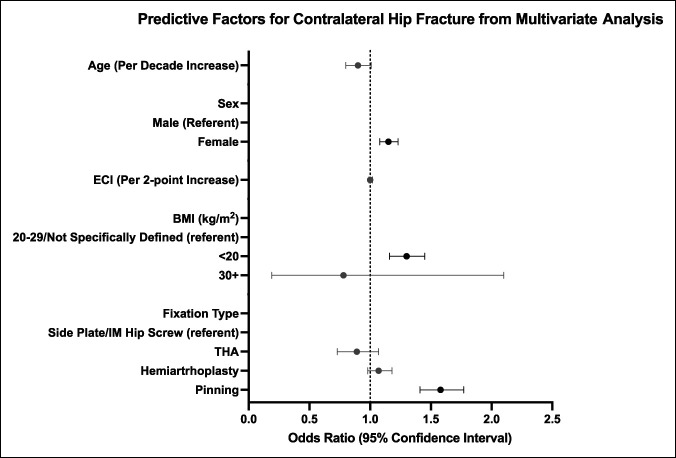
Forest plot showing independent predictors of contralateral hip fracture within 2 years of initial hip fracture from multivariate analysis. Bars represent 95% confidence interval. Black markers/error bars depict statistically significant predictors, and grey markers/errors bars represent nonsignificant variables.

The results of multivariate analysis for independent predictors of contralateral hip fracture within 2 years of initial hip fracture are listed in Table 2 and visually presented in Figure 3. Again, predictors were identified for this interval because nearly 70% of contralateral hip fractures occurred in the first 2 years after initial hip fracture. Independent predictors of 2-year contralateral hip fracture included female sex (OR 1.15, *P* < 0.0001), BMI <20 (compared with BMI 20-20/not specifically defined, OR 1.30, *P* < 0.0001), and percutaneous pinning surgery for initial hip fracture fixation (compared with side plate/intramedullary hip screw, OR 1.58, *P* < 0.0001). On multivariate analysis, the risk of 2-year contralateral hip fracture was not associated with age or ECI.

**Table 2:** Multivariate Analysis of Predictive Factors for Contralateral Hip Fracture Within 2 Years of Initial Hip Fracture

N = 104,311	OR (95% CI)	*P*
Age (per decade increase)	0.90 (0.80-1.01)	0.0777
Sex		
Male (referent)		
Female	1.15 (1.08-1.23)	**<0.0001**
ECI (per 2-point increase)	1.00 (0.99-1.02)	0.7918
BMI (kg/m^2^)		
<20	1.30 (1.16-1.45)	**<0.0001**
20-29/not specifically defined (referent)		
30+	0.78 (0.19-2.10)	0.6682
Fixation type		
Pinning	1.58 (1.41-1.77)	**<0.0001**
Side plate/IM hip screw (referent)		
Hemiarthroplasty	1.07 (0.98-1.18)	0.1194
THA	0.89 (0.73-1.07)	0.2321

BMI = body mass index, CI = confidence interval, ECI = Elixhauser Comorbidity Index, OR = odds ratio. Bold *P* values indicate statistical significance.

## Discussion

Geriatric fragility hip fractures present a major public health concern and are associated with high morbidity, mortality, and healthcare costs.^19,20^ Although ample evidence exists regarding the high incidence of hip fracture in the geriatric population, relatively little prior literature explores the risk of contralateral hip fracture after initial hip fracture^21,22^—an area of considerable interest for improving patient care and reducing healthcare costs. Thus, this study explores the incidence of and predictive factors for contralateral hip fracture using a large national health administrative database.

Of the 104,311 patients with geriatric hip fracture identified, 7,186 (6.9%) experienced a contralateral hip fracture in the 10 years after their initial hip fracture, with nearly 70% of all contralateral fractures occurring in the first 2 years. Accounting for loss of follow-up because of patient death or other factors, the 10-year contralateral hip fracture incidence nears 13%. Previous smaller studies have reported relatively similar rates of contralateral hip fracture in geriatric patients, with a 4-year incidence rate of 5.6%.^23^ Furthermore, large meta-analyses have reported an increased risk of contralateral hip fracture in the early years after initial hip fracture.^23,24^ Given these findings, there is a substantial risk of contralateral hip fracture after initial hip fracture, particularly soon after. Patient counseling, physical therapy, pharmacological treatment, and other preventive measures might minimize the risk of subsequent fracture in those patients at greatest risk—particularly in the acute period after initial hip fracture.^25-27^

Independent predictors of contralateral hip fracture were then explored with focus on the 2-year after index fracture because this was found to be the period of greatest risk and less confounded by loss from the database. Female sex was a notable independent predictor of contralateral hip fracture after initial hip fracture (OR 1.15). Given the markedly higher risk of osteoporosis in female patients, particularly in those older than 65 years, the greater risk of contralateral hip fracture in geriatric female patients is presumably logical.^28^ Osteoporosis has repeatedly been identified as one of the most important risk factors of geriatric fragility fracture.^29^ Given this, female patients who experience initial hip fracture could be even more closely followed for osteoporosis, appropriately treated, and counseled on their potentially increased risk of subsequent hip fracture.

Low BMI (<20) was found to be a notable independent risk factor of contralateral hip fracture (OR 1.30). Previously, studies have similarly reported that poor nutrition and low BMI are notable predictors of fragility hip fracture and subsequent fragility hip fracture.^30,31^ The protective effect of increased body weight on fractures may be because of greater mechanical loading on bone and higher serum levels of estrogen and adipokines, which contribute to increased bone mineral density.^32^ Given this finding, geriatric patients with lower BMI who experience hip fracture should be appropriately counseled on their increased risk of subsequent fracture, with additional precautionary measures organized through discussions between patients, providers, and patient support systems.

Regarding hip fracture fixation surgery, compared with the most commonly conducted fixation procedure (side plate/intramedullary hip screw), patients who had percutaneous pinning fixation done for their initial hip fracture were at markedly increased risk of contralateral hip fracture (OR 1.58). Percutaneous pinning has generally been indicated for nondisplaced fractures of the femoral neck because of comparatively low invasiveness and risk of postoperative complications compared with hemiarthroplasty, sliding hip screw, or other fixation methods.^33,34,35,36^ Additional investigation is required to better understand why patients who undergo percutaneous pinning may be at greater risk of subsequent contralateral hip fracture.

Hip fractures are often considered to be one of the most pressing public health concern faced by orthopaedic surgeons—with an estimated lifetime incidence of up to 18% in female and 6% in male patients.^22^ Given the high morbidity and mortality associated with geriatric hip fractures, it is of great interest for orthopaedic surgeons to reduce the incidence of subsequent contralateral hip fractures after primary hip fracture. The large, representative study population afforded by the currently used data set provided additional statistical power and ability to correlate with independent predictive factors.

This study is not without limitations. For one, as with any study using administrative data, it is reliant on and, therefore, limited to the accuracy of the coded administrative data. Despite this, the incidence of hip fracture and hip fracture fixation would be thought to be accurately captured within the database. Furthermore, given that the patient population studied was older and undergoing a high morbidity/mortality event (hip fracture), attrition bias secondary to losing patients to death or other factors posed a notable limitation during the 10-year follow-up period. Despite this, the 10-year Kaplan-Meier survival analysis should provide a better estimate of the overall incidence of contralateral hip fractures when accounting for attrition bias.

In sum, of 104,311 index geriatric fragility hip fracture patients identified in the data set, contralateral hip fracture within 10 years of their initial hip fracture was noted to be 12.9% by Kaplan-Meier survival analysis. Nearly 70% of all contralateral hip fractures occurred in the first 2 years after initial fracture, with the incidence being approximately 3 times greater in the first year than in the second year. Independent predictors of contralateral hip fracture included female sex, low BMI (<20 kg/m^2^), and percutaneous pinning for initial fixation surgery. Through better understanding of the incidence, timing, and risk factors of contralateral hip fracture, care pathways can be modified to reduce patient risk, particularly among those most vulnerable.
